# Cellular DNA content--a stable feature in epithelial ovarian cancer.

**DOI:** 10.1038/bjc.1984.29

**Published:** 1984-02

**Authors:** M. L. Friedlander, I. W. Taylor, P. Russell, M. H. Tattersall

## Abstract

Detailed flow cytometric analysis of cellular DNA content was performed on neoplastic tissue from 33 patients with malignant common epithelial ovarian tumours in order to investigate the intratumoral stability of ploidy and proliferative fraction. There was a remarkable stability, both spatial and temporal, in the DNA pattern for any particular tumour. Of 24 tumours that were analysed in multiple areas tumour ploidy was found to be a stable marker in all but 3 cases where regional variations were evident. In 9 patients serial analyses were performed on tumour obtained either at initial diagnosis (6 patients) or second look laparotomy (3 patients) and then some time later (7-17 months) at relapse or death and in all cases the tumour ploidy remained unchanged. In addition, 10 ovarian carcinomas established in nude mice have maintained a DNA content during serial passage similar to that of the original implanted tumour. In contrast in 50% of tumours that were evaluable for S-phase analysis we demonstrated a considerable intratumoral variability in the S-phase fraction. We conclude that cellular DNA content is a stable feature of ovarian carcinoma while S-phase fraction is commonly subject to intratumoral variation.


*Br. J. Cancer* (1984), **49**, 173–179

### Cellular DNA content—a stable feature in epithelial ovarian cancer

M.L. Friedlander[1], I.W. Taylor[1], P. Russell[2] and M.H.N. Tattersall[1]

[1]*Ludwig Institute for Cancer Research (Sydney Branch), Blackburn Building, University of Sydney, Sydney, N.S.W. 2006 and* [2]*Department of Anatomical Pathology, King George V Memorial Hospital, Camperdown, N.S.W. 2050, Australia.*

Detailed flow cytometric analysis of cellular DNA content was performed on neoplastic tissue from 33 patients with malignant common epithelial ovarian tumours in order to investigate the intratumoral stability of ploidy and proliferative fraction. There was a remarkable stability, both spatial and temporal, in the DNA pattern for any particular tumour. Of 24 tumours that were analysed in multiple areas tumour ploidy was found to be a stable marker in all but 3 cases where regional variations were evident. In 9 patients serial analyses were performed on tumour obtained either at initial diagnosis (6 patients) or second look laparotomy (3 patients) and then some time later (7–17 months) at relapse or death and in all cases the tumour ploidy remained unchanged. In addition, 10 ovarian carcinomas established in nude mice have maintained a DNA content during serial passage similar to that of the original implanted tumour. In contrast in 50% of tumours that were evaluable for S-phase analysis we demonstrated a considerable intratumoral variability in the S-phase fraction.

We conclude that cellular DNA content is a stable feature of ovarian carcinoma while S-phase fraction is commonly subject to intratumoral variation.

The flow cytometric determination of cellular DNA content has been shown to be of prognostic value in a number of tumour types (Bunn *et al.*, 1982; Wolley *et al.*, 1982) and we have recently reported that tumour ploidy was an independent prognostic variable and the major determinant of survival in patients with advanced ovarian cancer (Friedlander *et al.*, 1983*a*). The value of a single estimation of tumour ploidy would be clearly limited however if tumours commonly exhibited a variation in ploidy within different regions of the primary tumour or its metastases. Such variability has been reported in colonic cancer (Petersen *et al.*, 1981) and small cell lung cancer (Vindelov *et al.*, 1980) but there are no studies that have addressed the stability of cellular DNA content as determined with flow cytometry in ovarian tumours. We report the results of a study investigating the frequency of intratumoral heterogeneity of cellular DNA content and proliferative fraction (S-phase) in ovarian carcinomas.

### Materials and methods

Tumour specimens for flow cytometric analysis and histological examination were obtained from 33 patients with ovarian carcinoma and multiple areas (mean 4, range 2–12) were biopsied from either the primary tumour alone (8 FIGO I and II tumours) or from the metastatic sites as well when the patient had advanced stage disease (16 patients). Particular attention was paid to include biopsies from the peripheral and central regions of a tumour mass when possible. In an additional 9 patients it was possible to analyse tumour ploidy sequentially on specimens obtained at either initial diagnosis (6 cases) or from residual tumour found at second look laparotomy (3 cases) and then at subsequent relapse. Operative specimens were received fresh from the operating theatres and tumour tissue was subdivided in the pathology department for flow cytometry and histological examination. DNA flow cytometry was usually performed within 2 h of operative removal of tumour from the patient but if not, samples were stored at −70°C prior to analysis. Ascitic or pleural fluid specimens were examined cytologically and flow cytometrically immediately following aspiration.

Correspondence: M.L. Friedlander.
Received 5 September 1983; accepted 10 October, 1983.

### *Flow cytometry*

*DNA content* All specimens were analysed on an ICP 22 flow cytometer (Ortho-Instruments, Westwood, MA). Specimens were processed and stained for DNA content with ethidium bromide and mithramycin in a single step staining technique that has been fully described elsewhere (Taylor, 1980). Briefly, for solid tumours 250 $\mu l$ of a 1% Triton-x-100 solution containing 400 $\mu g\,ml^{-1}$ ethidium bromide and 250 $\mu l$ of a 1% triton solution containing 125 $\mu g\,ml^{-1}$ mithramycin and 75 mM magnesium chloride were added to 2 ml of RPMI

© The Macmillan Press Ltd., 1984

1640 medium containing 10% foetal calf serum. A 2 ml aliquot of this staining solution plus 200 $\mu$l chicken red blood cells (CRBC; $10^6 ml^{-1}$) were added to a small piece of tumour tissue (5 mm x 5 mm) in a petri dish and the tissue was finely disaggregated with scalpel blades. A further 2 ml of staining solution were added, the solution gently pipetted several times and the preparation filtered through an 80 $\mu$m nylon mesh filter. This preparation results in a suspension of isolated nuclei from which almost all cytoplasm has been removed. RNA-ase (ribonuclease Type IA, Sigma Chemical Company) was added to the suspension just prior to analysis to give a final concentration of 1 mg $ml^{-1}$.

Chicken red blood cells (CRBC) were used as an internal marker, as the ratio of the $G_1$ DNA content of human diploid cells to the DNA content of CRBC is highly reproducible ($2.9 \pm 0.17$) under the staining conditions used (Taylor & Milthorpe, 1980). Using this standard all the tumour specimens analysed contained a population of cells with a diploid DNA content. When this was the only cell population present the tumour was classified as diploid, while tumours which had evidence of an additional $G_1$ peak were classified as aneuploid. Where in addition to the diploid $G_1$ peak there was clear evidence of more than one aneuploid $G_1$ peak the tumour was classified as multiploid. Ploidy was further quantitated by the DNA Index (DI) which represents the relative DNA content of the aneuploid $G_1$ cells in comparison to diploid cells (Barlogie *et al.,* 1980). Thus, a DI of 1 is synonymous with a normal diploid DNA content. The mean coefficient of variation of the diploid or aneuploid $G_1$ peak was 2.7% in all tumours studied (range 1.2–5.1).

In order to determine the sensitivity of the method of detecting an aneuploid tumour population, normal human lymphocytes (diploid) were admixed with an aneuploid ovarian cell line (DI = 1.59). Varying amounts of aneuploid cells were mixed with human lymphocytes to give a final cell concentration of $10^6 ml^{-1}$ with an aneuploid cell content ranging from 0.5%–95%. The cells were stained with ethidium bromide and mithramycin as described above.

*Proliferative fraction* The proportion of cells in S-phase was estimated by computer analysis in 16 tumours using a planimetric method of analysis (Milthorpe, 1980). Overlapping of subpopulations was the major reason for omitting tumour samples from analysis and while all diploid tumours could be analysed, in only 50% of tumours with aneuploid cells was S-phase analysis attempted.

*Xenografted tumours*

Balb/C nu/nu strain female nude mice were inoculated with fragments of ovarian tumour tissue as previously described (Van Haaften–Day *et al.,* 1983). All tumours had DNA analysis and histological examination performed prior to implantation and with each serial passage.

### Results

*Comparison of tumour ploidy within primary tumour and metastases*

Twenty-four patients had tumour ploidy determined either from different sites within the primary tumour alone (8 cases) or from the metastatic sites as well in those patients with advanced stage disease. Thirty-three percent of the patients had diploid tumours and 67% had aneuploid tumours. Figure 1 outlines the distribution of DNA content and demonstrates a clustering of tumour cells about a diploid and triploid-tetraploid mode. The majority of tumours (87%), exhibited a stable DI in all sites analysed (Figure 2) and this regional stability of DNA content was also evident in 2 multiploidal tumours (Figure 3). The 3 exceptions (all Stage III tumours) included a tumour which was initially classified as diploid but had an additional tetraploid $G_1$ peak demonstrated in one region and two aneuploid tumours which showed regional variations of aneuploidy (Figure 4). Only one of these tumours had concomitant regional histological differences which correlated with the ploidy variations (a mixed epithelial carcinoma with adenosquamous and typical serous carcinoma in different areas).

Although the DI was stable in different areas of most tumours there were nevertheless often regional variations (for examples, see Figure 4) in the actual proportion of aneuploid to diploid cells. We determined the sensitivity of flow cytometry in detecting aneuploid populations and under controlled conditions demonstrated that a 1% concentration of aneuploid cells admixed with normal human lymphocytes could be identified (Figure 5).

*Sequential analysis of DNA content*

Nine patients had DNA analysis performed on sequential tumour specimens. The initial specimen was obtained either at initial diagnosis or at a second look laparotomy where residual tumour remained after chemotherapy. Tumour DNA analysis was repeated in all patients at a subsequent relapse or death with a mean interval of time

**Figure 1** Frequency histogram showing the distribution of ploidy in tumours from 24 patients with ovarian cancer. Ploidy is expressed as DNA index (DI) which represents the relative DNA content of tumour $G_1$ to diploid $G_1$ cells. All tumours analysed contained a population of cells with a diploid DNA content and when this was the only cell population present the tumour was classified as diploid (DNA index 1). When there was an additional $G_1$ peak the tumour was classified as aneuploid and in such cases the diploid component was assumed to be made up of normal cells. Five tumours had 2 aneuploid $G_1$ peaks while the rest had a single tumour $G_1$ peak.

between the first and second DNA analysis of 9 months (range, 7–15 months). Two patients initially had diploid tumours and 7 had aneuploid tumours and the DI of all tumours remained unchanged at the time of relapse or death (Table I).

*Xenografted ovarian tumours*

Ten epithelial ovarian carcinomas were established directly in nude mice and histological examination and DNA analyses were performed on tumour tissue just prior to implantation and repeated at each subsequent passage. The DI (Table II) and histological features have remained essentially unchanged during the serial passage of all tumours to date (median time = 12 months).

**Table I** Comparison of DNA index at diagnosis and at time of relapse or death

| *Patient* | *Initial DNA index* | *DNA index at relapse* |
|---|---|---|
| 1 | 0.85 | 0.83 |
| 2 | 0.9 | 0.85 |
| 3 | 1 | 1 |
| 4 | 1 | 1 |
| 5 | 1.17 | 1.15 |
| 6 | 1.31 | 1.31 |
| 7 | 1.73 | 1.73 |
| 8 | 1.78 | 1.77 |
| 9 | 1.83 | 1.79 |

**Table II** Comparison of DNA index of ovarian tumour xenografts passaged in nude mice with the DNA index of original tumour

| *Tumour* | *Original DI of fresh tumour* | *DI after passage in nude mice* | *No. of passages* |
|---|---|---|---|
| 1 | 1.64 | 1.73 | 4 |
| 2 | 2.60 | 2.53 | 2 |
| 3 | 0.86 | 0.92 | 6 |
| 4 | 1.45 | 1.60 | 4 |
| 5 | 1.35 | 1.40 | 3 |
| 6 | 1.33 | 1.42 | 3 |
| 7 | 2 | 2 | 5 |
| 8 | 1.47 | 1.52 | 6 |
| 9 | 1 | 1 | 3 |
| 10 | 1.72 | 1.9 | 3 |

**Figure 2** Histogram to demonstrate the relative regional stability of tumour ploidy and relative regional variation of S-phase fraction in 24 ovarian cancers sampled in different areas of the tumour. Apart from 1 predominantly diploid tumour which had evidence of an additional tetraploid component in one area and 2 aneuploid tumours which exhibited regional variations of aneuploidy tumour ploidy was a stable marker. S-phase analysis was possible in all diploid tumours but only in 50% of aneuploid tumours as overlapping of cell populations precluded accurate analysis. A 40% or greater variation of S-phase was evident within different areas of the same tumour in half the cases analysed.

**Figure 3** Demonstration of stability of DNA content in a multiploid tumour. There is evidence of 2 aneuploid clones in both the primary tumour and omental metastases. Note the resolving power of the instrument in discriminating the 2 diploid peaks. Channel no., relative fluorescence intensity (DNA content). Ordinate, number of cells.

### *Regional S-phase variation*

Of the 24 tumours studied for regional variations in DNA content all 8 diploid tumours were evaluable for S-phase analysis while overlapping of cell populations precluded an S-phase estimation in half the tumours with aneuploid cells. Diploid tumours had a mean S-phase of 6.6% (range, 3.4–16.2%) and aneuploid tumour cells a mean S-phase of 17.3% (range, 3.7–33.6%). In 8 of the tumours (50%) there was a 40% or greater variation of S-phase (Figure 2) in different sites.

### Discussion

Flow cytometric analysis allows tumour DNA content and proliferative activity to be determined rapidly and precisely and their value as possible objective parameters reflecting tumour biology is of intense interest. We have shown that cellular DNA content is an independent prognostic variable and a major determinant of survival in advanced ovarian cancer (Friedlander *et al.*, 1983*a*) and similar findings have been reported in other malignancies (Bunn *et al.*, 1982; Wolley *et al.*, 1982). There is however, some evidence that regional variations in tumour ploidy are common in colonic carcinomas (Petersen *et al.*, 1981) and small cell lung cancer (Vindelov *et al.*, 1980) and the heterogeneity of such tumours casts doubts on the value of a single estimation of ploidy as a prognostic index for all tumour types. We have therefore studied the stability of cellular DNA content in common epithelial ovarian cancer.

While ovarian tumours as a group are widely heterogeneous with respect to DNA content (Atkin, 1970; Friedlander *et al.*, 1983*b*) with early stage ovarian cancers being commonly diploid and

**Figure 4** DNA histograms of a tumour that exhibited a regional variation in aneuploidy. *Channel number* represents fluorescence intensity which is directly proportional to DNA content. The numbef of cells counted are shown on the ordinate. The first peak corresponds to chicken red blood cells (CRBC) which acts as an internal biological standard and is set in channel 10. The next major peak represents the diploid $G_1$ peak. Note that in subset c only one aneuploid population is evident while in subsets a and b two aneuploid populations are present but the relative proportions vary.

advanced stage carcinomas commonly aneuploid, we demonstrate in this study that within an individual tumour intratumoral variations of ploidy are uncommon, occurring in only 13% of cases. These findings support those of an earlier study where, using Feulgen microspectrophotometry, ovarian cancers were shown to have a stable DNA content within different sites (Atkin, 1970). Further evidence of the stability of cellular DNA content in epithelial ovarian cancer is provided by finding that in ovarian tumours established in nude mice the DI remains unchanged during serial passage. The mean variation of the DI before and after passage in nude mice was 4.5% (range, 0–10%). This is in keeping with the variation that can be expected by possible staining and instrumental variation (Taylor & Milthorpe, 1980).

It was possible to determine the stability of cellular DNA content during tumour progression in 9 patients by analysing tumour specimens obtained at initial diagnosis or at a second look laparotomy and then again 7–17 months later at the time of relapse or death and in all cases the DI remained unchanged. All these patients had initially responded to chemotherapy with *cis*-platinum and/or chlorambucil and had later relapsed. It is interesting that there was a reappearance of clones with the same DNA content as that found prior to treatment, which suggests some stability of the genome. There could however be chromosomal variability associated with disease progression but masked either by minor changes undetectable with flow cytometry (Barlogie *et al.,* 1977) or because chromosomal number variability may be due to a DNA packaging defect which would not be reflected by a change in the DNA content (Kraemer *et al.,* 1971). Only a small number of cases have been reported where serial karyotyping and banding studies have been performed following the progression of ovarian tumours and such studies show that the karyotype remains basically unchanged during tumour progression with changes, when present, representing a variation in the theme already observed in the original tumours (Atkin, 1970; MacKillop *et al.,* 1983; Sandberg, 1982).

While the DI remains constant within a primary tumour and its metastases the proportion of aneuploid cells often varies from one region to another. This feature has been interpreted by some (Nervi *et al.,* 1982) as evidence of intratumoral heterogeneity but it is likely that these variations reflect different degrees of admixing of normal diploid cells with aneuploid tumour cells rather than changes of biological significance. We cannot rule out entirely the possibility that cells with a diploid DNA content in tumours with bimodal

**Figure 5** Determination of the sensitivity of flow cytometry in detecting an aneuploid cell population. Varying concentrations of aneuploid cells were admixed with human lymphocytes (diploid) to determine the sensitivity of detection of aneuploidy. A 1% or greater content of aneuploid cells could be detected particularly by collecting up large numbers of cells and amplifying the vertical scale. The broken line represents a 100 × magnification of an aneuploid population. Channel number, relative fluorescence intensity (DNA content). Ordinate, number of cells. The major $G_1$ peak represents diploid lymphocytes and the smaller peak at channel number 70 the aneuploid population.

DNA distributions could represent an additional tumour clone but a number of studies have shown that in these circumstances the diploid cells usually represent normal cells (Barlogie *et al.*, 1978; Perez *et al.*, 1981).

We determined the sensitivity of flow cytometry to detect aneuploid tumour cells admixed with normal cells under controlled conditions and showed that as low as a 1% content of tumour cells could be detected. However, for practical purposes, we suggest the limit of detection of an aneuploid cell population is between 5% and 10% of the cells analysed due to the presence of background debris and the possible masking effect of the S-phase and $G_2$+M components of diploid cells. Histological examination of all specimens analysed is therefore necessary to ensure that they contain adequate and representative portions of tumour tissue. This assumes particular importance in diploid tumours where the finding may have important prognostic implications, as these tumours have a more favourable natural history than aneuploid tumours (Atkin, 1970; Bunn *et al.*, 1982, Friedlander *et al.*, 1983*b*; Wolley *et al.*, 1982). The actual degree of aneuploidy (i.e. DI of tumour) or the presence of multiploidy has not been demonstrated to date to be of significance and although it has been suggested by some that multiploid tumours may have a more aggressive natural history (Taylor *et al.*, 1983; Vindelov *et al.*, 1980), we could not confirm this in patients with advanced ovarian cancer. All 3 patients who had regional variations in ploidy in this study relapsed within 6 months of diagnosis and it is possible that an unstable DNA content identifies high risk patients.

The S-phase as determined flow cytometrically has been reported to be a reflection of the aggressiveness of tumour behaviour and to be of prognostic significance (Costa *et al.*, 1981). The S-phase can be determined in all diploid tumours but only in ~50% of aneuploid tumours because of overlapping of tumour populations. While we and others have demonstrated that diploid tumours have a lower S-phase than aneuploid tumours, it is possible that the values for S-phase in diploid cells have been artificially lowered by the presence of normal non-cycling diploid cells e.g. lymphocytes (Dosik *et al.*, 1980), while aneuploid tumours could have a higher S-phase because of the presence of diploid doublets and tetraploid normal cells. In this study we found that up to 50% of tumours showed a significant regional variation in the S-phase fraction (>40% variation in different areas). The reasons for this variability are not known but could relate either to different proliferative states among tumour cells in different nutritional environments, or to variable contamination of tumour cells by normal non-cycling cell populations. The value of a single S-phase estimation is therefore clearly limited in the light of these findings, as are data relating to the prognostic value of S-phase in ovarian cancer.

We conclude that DNA content is a stable feature in most cases of epithelial ovarian cancer exhibiting constancy within the primary tumour, its metastases and during subsequent tumour progression. On the other hand, the S-phase fraction is often subject to considerable intratumoral variation. These results cannot necessarily be extrapolated to other tumour types where similar studies are required to establish the representative value of a single estimation of tumour DNA content or proliferative fraction.

### References

ATKIN, N.B. (1970) Modal DNA value and chromosome number in ovarian neoplasia. *Cancer* **27**, 1064.

BARLOGIE, B., DREWINKO B., SCHUMANN, J. & 5 others (1980) Cellular DNA content as a marker of neoplasia in man. *Am. J. Med.*, **69**, 195.

BARLOGIE, B., GOHDE, W., JOHNSTON, D.A. & 4 others (1978). Determination of ploidy and proliferative characteristics of human solid tumours by pulse cytophotometry. *Cancer Res.*, **38**, 3333.

BARLOGIE, B., HITTELMAN, W., SPITZER G. & 4 others (1977). Correlation of DNA distribution abnormalities with cytogenetic findings in human adult leukaemia and lymphoma. *Cancer Res.*, **37**, 4400.

BUNN, P.A., KRASNOW, S., MAKUCH, R.W., SCHLAM M.L. & SCHECTER, G.P. (1982). Flow cytometric analysis of DNA content of bone marrow cells in patient with plasma cell myeloma: clinical implications. *Blood*, **59**, 528.

COSTA, A., MAZZINI, G., DELBINO, G. & SILVESTRINI, R. (1981). DNA content and kinetic characteristics of non-Hodgkins lymphoma: determined by flow cytometry and autoradiography. *Cytometry*, **2**, 185.

DOSIK, G.M., BARLOGIE, B., GOHDE, W., JOHNSTON, D., TEKELL, J.L. & DREWINKO, B. (1980). Flow cytometry of DNA content in human bone marrow: a critical reappraisal. *Blood*, **55**, 734.

FRIEDLANDER, M.L., HEDLEY, D.W., TAYLOR, I.W., RUSSELL, P., COATES, A.S. & TATTERSALL, M.H.N. (1983*a*). The influence of cellular DNA content on survival in advanced ovarian cancer. *Cancer Res.* (In press).

FRIEDLANDER, M.L., TAYLOR, I.W., RUSSELL, P., MUSGROVE, E.A., HEDLEY, D.W., TATTERSALL, M.H.N. (1983*b*). Ploidy as a prognostic factor in ovarian cancer. *Int. J. Gynecol. Pathol.*, **2**, 55.

KRAEMER, P.M., PETERSEN, D.F. & VAN DILLA, M.A. (1971). DNA constancy in heteroploidy and the stem line theory of tumours. *Science*, **174**, 714.

MACKILLOP, W.J., TRENT, J.M., STEWART, S.S. & BUICK, R.N. (1983). Tumour progression studied by analysis of cellular features of serial ascitic ovarian carcinoma tumours. *Cancer Res.*, **43**, 874.

MILTHORPE, B.K. (1980). FMFPAKI: A program package for routine analysis of single parameter flow microfluorimetric data on a low cost mini computer. *Comput Biomed Res.*, **13**, 417.

NERVI, C., BADARACCO, G., MAISTO, A., MAURO, F., DONATELLA, T.P., STARACE, G. (1982). Cytometric evidence of cytogenetic and proliferative heterogeneity of human solid tumours. *Cytometry*, **2**, 303.

PEREZ, D.J., TAYLOR, I.W., MILTHORPE, B.K., MCGOVERN, V.J. & TATTERSALL, M.H.N. (1981). Identification and quantitation of tumour cells in cells suspensions: a comparison of cytology and flow cytometry. *Br. J. Cancer*, **43**, 526.

PETERSEN, S.E., LORENTZEN, M., BICHEL P. (1981). A mosaic subpopulation structure of human colorectal carcinomas demonstrated by flow cytometry. *Acta Pathol. Microbiol. Scand.* (Suppl.) **274**, 412.

SANDBERG, A.A. (1982). Chromosomal changes in Human Cancers: Specificity and heterogeneity. In: *Tumour Cell Heterogeneity: Origins and Implications*, (Eds Owens *et al.*) Bristol Myers Cancer Symposia, V **4**, 370.

TAYLOR, I.W. (1980). A rapid single step staining technique for DNA analysis by flow microfluorimetry. *J. Histochem. Cytochem.*, **28**, 1021.

TAYLOR, I.W. & MILTHORPE, B.K. (1980). An evaluation of DNA fluorochromes, staining techniques and analysis for flow cytometry. *J. Histochem. Cytochem.*, **28**, 1224.

TAYLOR, I.W., MUSGROVE, E.A., FRIEDLANDER, M.L., FOO, M.S., HEDLEY, D.W. (1983). The influence of age on the DNA ploidy levels of breast tumours. *Eur. J. Cancer*, **19**, 623.

VAN HAAFTEN-DAY, C., RUSSELL, P., RUGG, C., WILLS, E.J. & TATTERSALL, M.H.N. (1983). Flow cytometric and morphological studies of ovarian carcinoma cell lines and xenografts. *Cancer Res.*, **43**, 3725.

VINDELOV, L.L., HANSEN, H.H., CHRISTENSEN, I.J. & 4 others (1980). Clonal heterogeneity of small cell anaplastic carcinoma of the lung demonstrated by flow cytometric DNA analysis. *Cancer Res.*, **40**, 4295.

WOLLEY, R.C., SCHREIBER, K., KOSS, L.A., KARAS, M. & SHERMAN, A. (1982). DNA distribution in human colon carcinomas and its relationship to clinical behaviour. *J. Natl. Cancer Inst.*, **69**, 15.


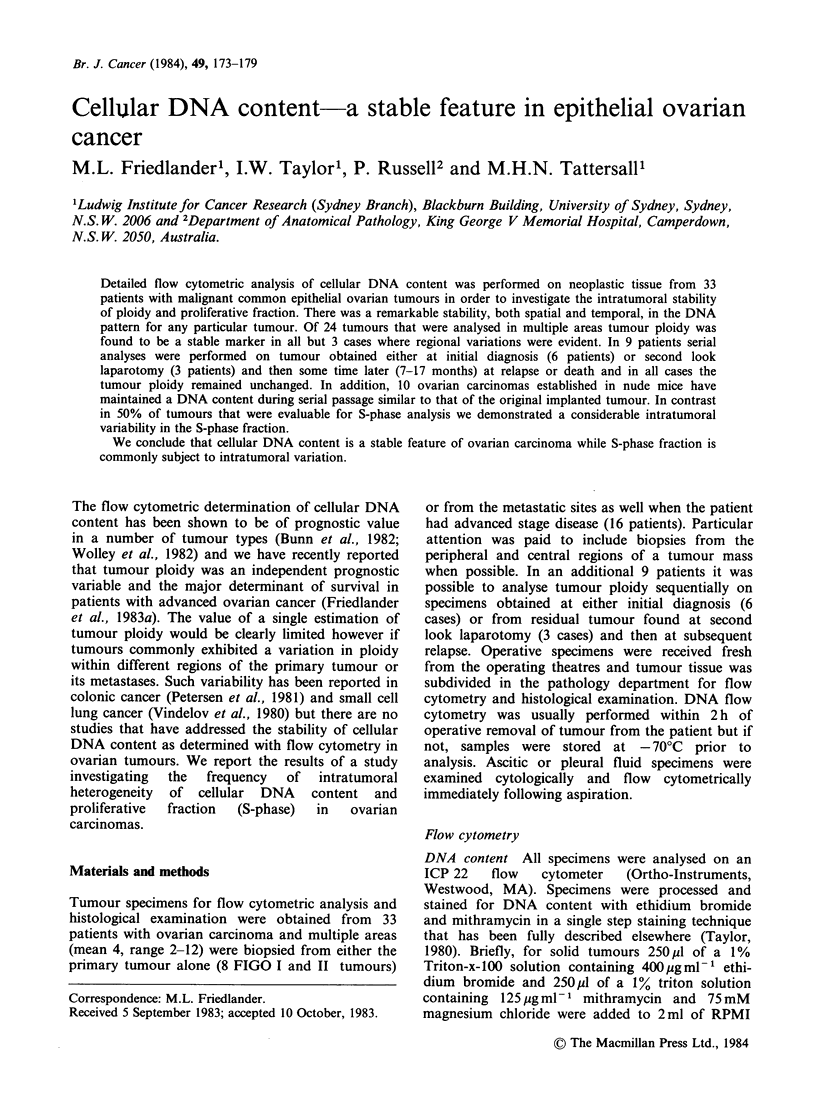

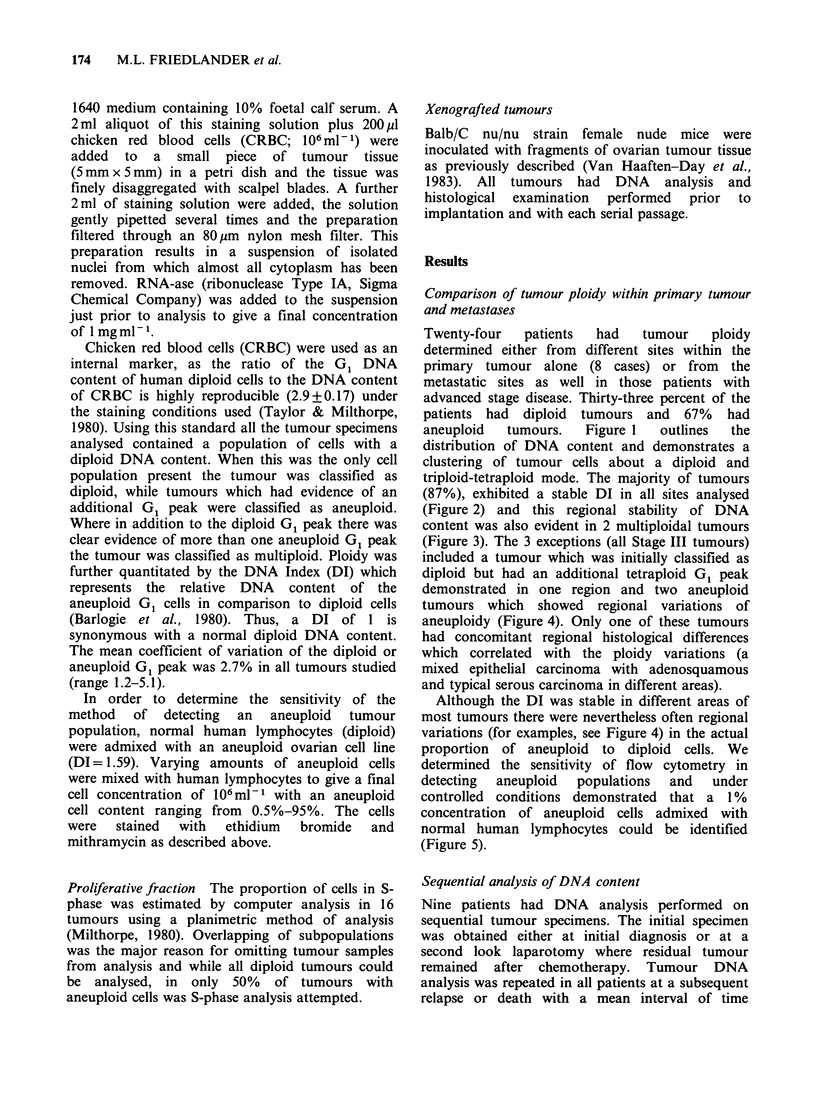

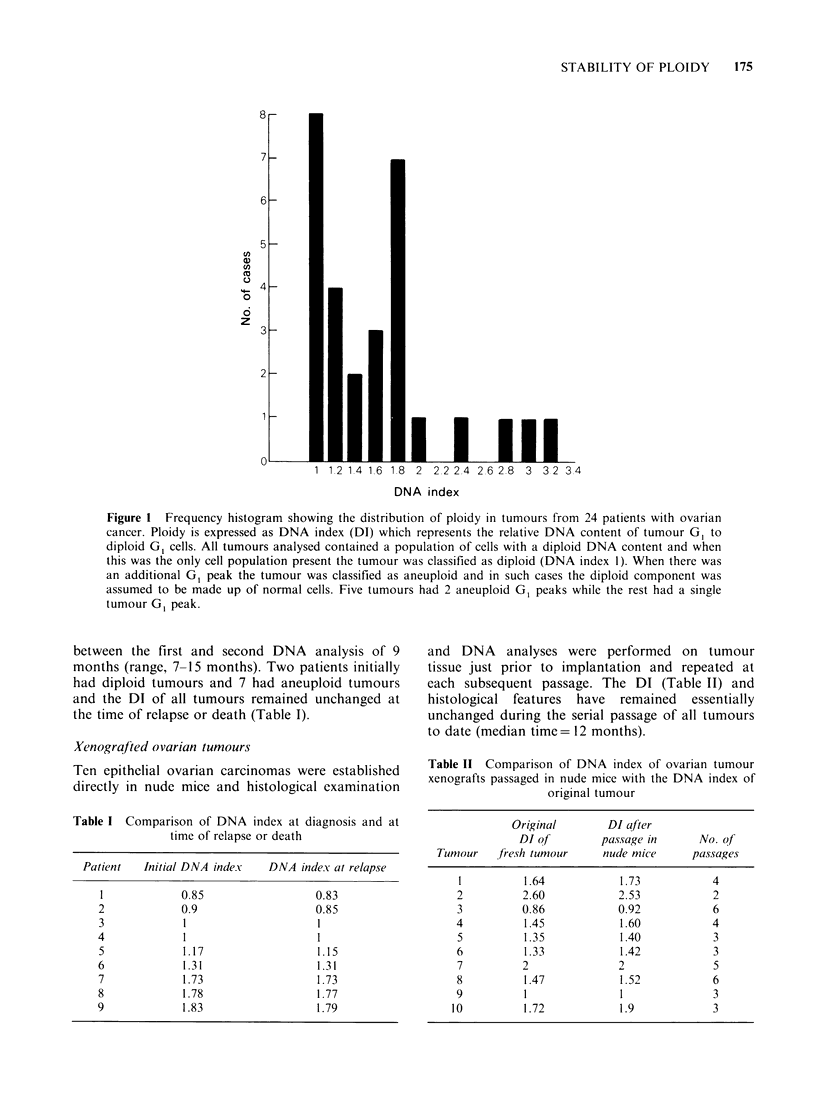

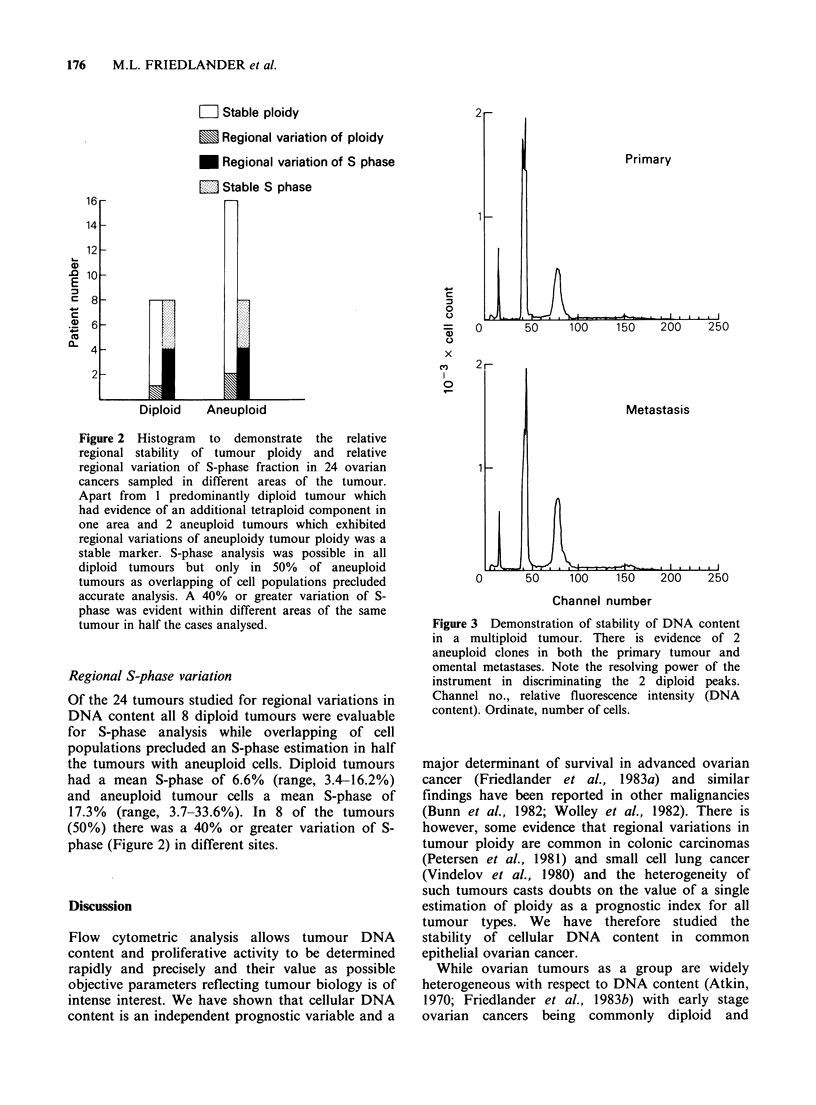

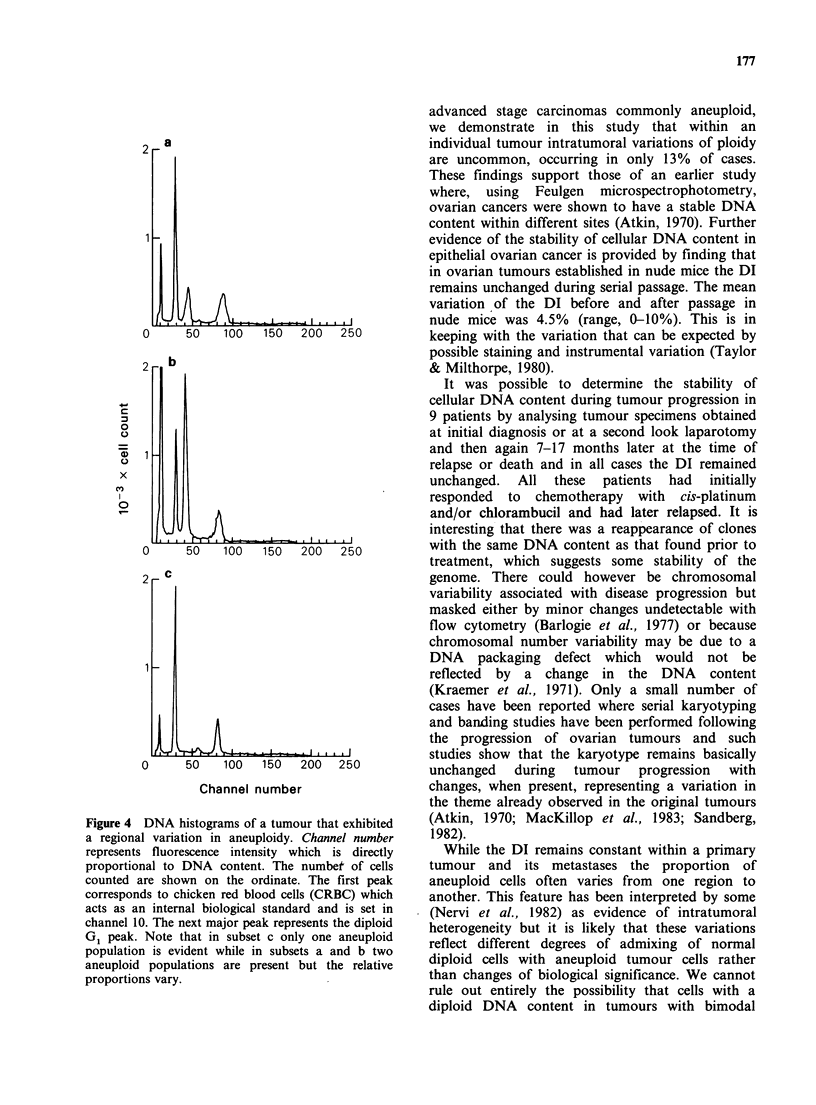

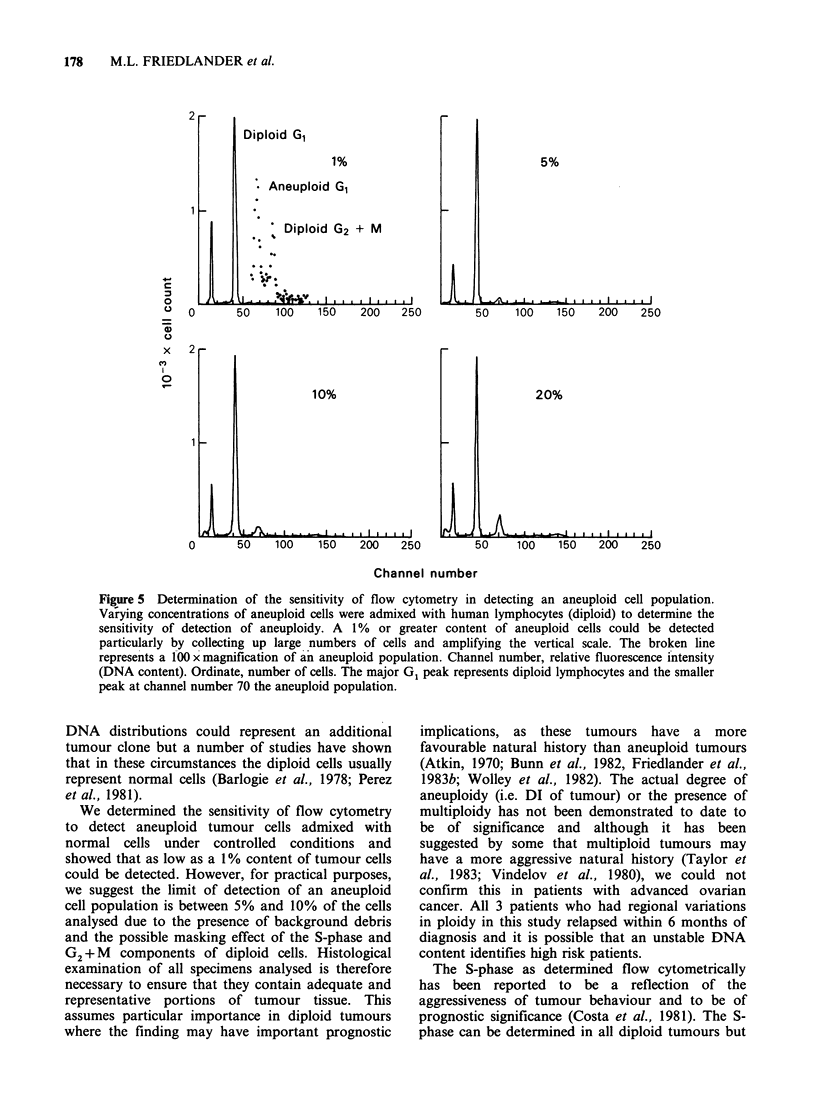

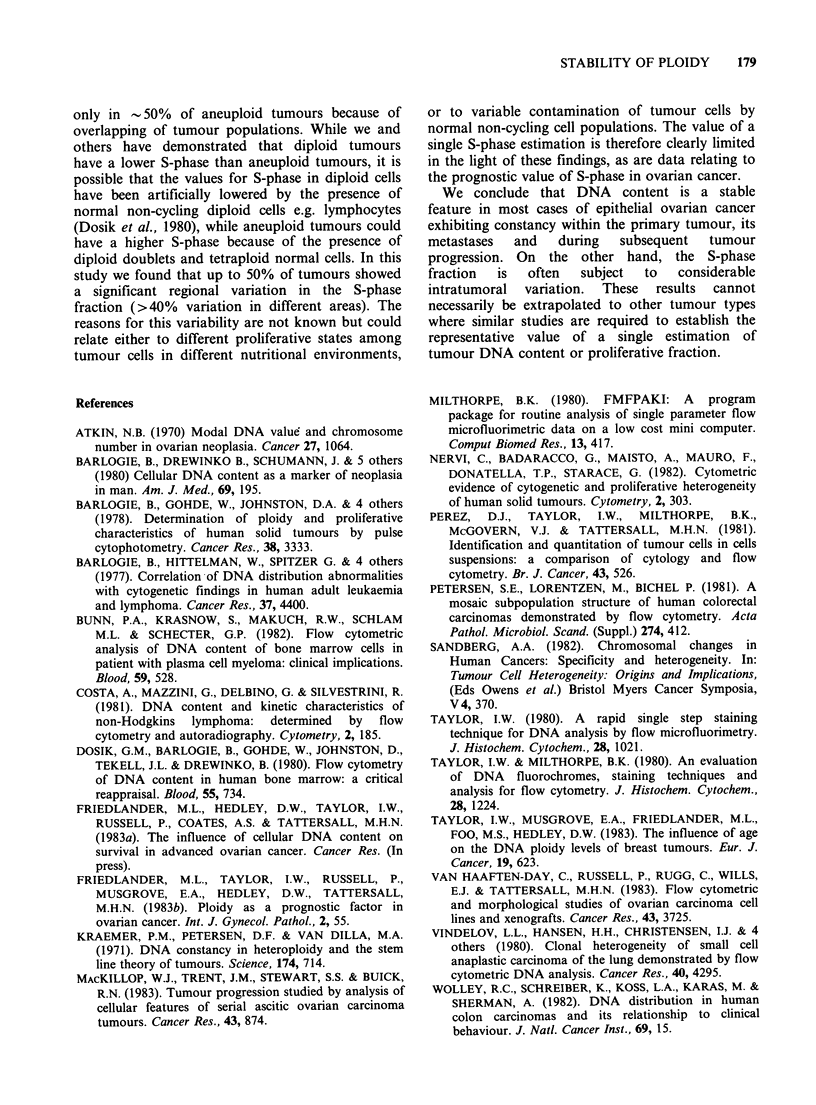


## References

[OCR_00775] Atkin N. B. (1971). Modal DNA value and chromosome number in ovarian neoplasia. A clinical and histopathologic assessment.. Cancer.

[OCR_00779] Barlogie B., Drewinko B., Schumann J., Göhde W., Dosik G., Latreille J., Johnston D. A., Freireich E. J. (1980). Cellular DNA content as a marker of neoplasia in man.. Am J Med.

[OCR_00784] Barlogie B., Göhde W., Johnston D. A., Smallwood L., Schumann J., Drewinko B., Freireich E. J. (1978). Determination of ploidy and proliferative characteristics of human solid tumors by pulse cytophotometry.. Cancer Res.

[OCR_00788] Barlogie B., Hittelman W., Spitzer G., Trujilo J. M., Hart J. S., Smallwood L., Drewinko B. (1977). Correlation of DNA distribution abnormalities with cytogenetic findings in human adult leukemia and lymphoma.. Cancer Res.

[OCR_00794] Bunn P. A., Krasnow S., Makuch R. W., Schlam M. L., Schechter G. P. (1982). Flow cytometric analysis of DNA content of bone marrow cells in patients with plasma cell myeloma: clinical implications.. Blood.

[OCR_00800] Costa A., Mazzini G., Del Bino G., Silvestrini R. (1981). DNA content and kinetic characteristics of non-Hodgkin's lymphoma: determined by flow cytometry and autoradiography.. Cytometry.

[OCR_00806] Dosik G. M., Barlogie B., Göhde W., Johnston D., Tekell J. L., Drewinko B. (1980). Flow cytometry of DNA content in human bone marrow: a critical reappraisal.. Blood.

[OCR_00819] Friedlander M. L., Taylor I. W., Russell P., Musgrove E. A., Hedley D. H., Tattersall M. H. (1983). Ploidy as a prognostic factor in ovarian cancer.. Int J Gynecol Pathol.

[OCR_00825] Kraemer P. M., Petersen D. F., Van Dilla M. A. (1971). DNA constancy in heteroploidy and the stem line theory of tumors.. Science.

[OCR_00830] Mackillop W. J., Trent J. M., Stewart S. S., Buick R. N. (1983). Tumor progression studied by analysis of cellular features of serial ascitic ovarian carcinoma tumors.. Cancer Res.

[OCR_00836] Milthorpe B. (1980). FMFPAK1: a program package for routine analysis of single parameter flow microfluorimetric data on a low cost mini-computer.. Comput Biomed Res.

[OCR_00842] Nervi C., Badaracco G., Maisto A., Mauro F., Tirindelli-Danesi D., Starace G. (1982). Cytometric evidence of cytogenetic and proliferative heterogeneity of human solid tumors.. Cytometry.

[OCR_00848] Perez D. J., Taylor I. W., Milthorpe B. K., McGovern V. J., Tattersall M. H. (1981). Identification and quantitation of tumour cells in cell suspensions: a comparison of cytology and flow cytometry.. Br J Cancer.

[OCR_00868] Taylor I. W. (1980). A rapid single step staining technique for DNA analysis by flow microfluorimetry.. J Histochem Cytochem.

[OCR_00873] Taylor I. W., Milthorpe B. K. (1980). An evaluation of DNA fluorochromes, staining techniques, and analysis for flow cytometry. I. Unperturbed cell populations.. J Histochem Cytochem.

[OCR_00879] Taylor I. W., Musgrove E. A., Friedlander M. L., Foo M. S., Hedley D. W. (1983). The influence of age on the DNA ploidy levels of breast tumours.. Eur J Cancer Clin Oncol.

[OCR_00892] Vindeløv L. L., Hansen H. H., Christensen I. J., Spang-Thomsen M., Hirsch F. R., Hansen M., Nissen N. I. (1980). Clonal heterogeneity of small-cell anaplastic carcinoma of the lung demonstrated by flow-cytometric DNA analysis.. Cancer Res.

[OCR_00897] Wolley R. C., Schreiber K., Koss L. G., Karas M., Sherman A. (1982). DNA distribution in human colon carcinomas and its relationship to clinical behavior.. J Natl Cancer Inst.

[OCR_00885] van Haaften-Day C., Russell P., Rugg C., Wills E. J., Tattersall M. H. (1983). Flow cytometric and morphological studies of ovarian carcinoma cell lines and xenografts.. Cancer Res.

